# Combined Approach of Chromatographic Fractionation and Raman Spectroscopy for Metabolite Profiling of *Enterobacter* spp. Supernatant

**DOI:** 10.3390/ijms27031564

**Published:** 2026-02-05

**Authors:** Elizaveta Denisova, Anastasia Avdyusheva, Vera Vasilieva, Elizaveta Tyshchuk, Polina Grebenkina, Andrey Korenevsky, Ivan Chelibanov, Vladimir Chelibanov, Areg Totolian, Lyudmila Kraeva, Dmitry Sokolov

**Affiliations:** 1 Saint-Petersburg Pasteur Institute, St. Petersburg 197101, Russia; liza.denisova9898@yandex.ru (E.D.); lizatyshchuk@yandex.ru (E.T.); grebenkinap@gmail.com (P.G.); a.korenevsky@yandex.ru (A.K.); ichelibanov@gmail.com (I.C.); totolian@pasteurorg.ru (A.T.); lykraeva@yandex.ru (L.K.); 2 Research Institute of Obstetrics and Gynecology Named After D.O. Ott, St. Petersburg 199034, Russia; avdyusheva04@mail.ru (A.A.); vasvera2109@gmail.com (V.V.); 3 «OPTEK» Joint Stock Company, St. Petersburg 199178, Russia; chelibanov@gmail.com; 4 Military Medical Academy Named After S.M. Kirov, 6 Akademika Lebedeva Street, Building 6, St. Petersburg 194044, Russia

**Keywords:** ESKAPE pathogens, bacterial secretome, Raman spectroscopy, chromatographic fractionation, virulence metabolites

## Abstract

The secretome of ESKAPE pathogens contains numerous bioactive molecules that play a key role in pathogenesis and the formation of an immunosuppressive microenvironment. However, analyzing this complex chemical composition presents significant methodological challenges. In this study, we propose a combined approach integrating chromatographic fractionation of cell-free supernatants with Raman spectroscopy to deconstruct the secretome of the clinically relevant Gram-negative pathogen—*Enterobacter* spp. Chromatographic separation of the *Enterobacter* spp. supernatant into seven fractions reduced spectral congestion and enabled identification of fraction 3 as having a unique metabolite profile, enriched in peptides (including tryptophan- and tyrosine-containing structures), nucleic acids, polysaccharides, and putative glutathione-like compounds. Notably, fraction 3 lacked markers of phenylalanine and sterol-like lipids, highlighting its distinct composition. Compared to conventional mass spectrometry and nuclear magnetic resonance, our hybrid strategy offers minimal sample preparation, preserves sample integrity for repeated analysis, avoids ionization bias, and is fully compatible with aqueous biological matrices—critical advantages for profiling labile or low-abundance metabolites in native secretomes. These findings demonstrate that the combination of preparative chromatography and Raman spectroscopy effectively resolves complex bacterial secretomes and identifies fractions potentially carrying key virulence or signaling functions.

## 1. Introduction

Infections caused by multidrug-resistant (MDR) microorganisms represent one of the most serious threats to global public health in the 21st century [1]. Of particular clinical importance are pathogens grouped under the acronym ESKAPE (*Enterococcus faecium*, *Staphylococcus aureus*, *Klebsiella pneumoniae*, *Acinetobacter baumannii*, *Pseudomonas aeruginosa*, and *Enterobacter* spp.), which dominate the landscape of nosocomial infections due to their ability to rapidly acquire resistance genes, form biofilms, and persist in hospital environments [1,2]. According to the updated 2024 WHO Bacterial Priority Pathogens List (BPPL), carbapenem-resistant *Acinetobacter baumannii* and carbapenem-resistant or third-generation cephalosporin-resistant Enterobacterales—which include *Klebsiella pneumoniae*, *Escherichia coli*, and *Enterobacter* spp.—are classified as “critical priority” pathogens [2]. In contrast, carbapenem-resistant *Pseudomonas aeruginosa* is now categorized as a “high priority” pathogen, reflecting evolving resistance trends and regional differences in treatment options [2].

The genus *Enterobacter* includes bacteria that frequently cause nosocomial infections in immunosuppressed patients [2,3]. Despite their clinical significance, the secretome of *Enterobacter* spp.—particularly its non-proteinaceous metabolites—has been studied considerably less than that of other ESKAPE pathogens.

The pathogenesis of infections caused by these bacteria is shaped not only by genetic determinants of antibiotic resistance but also by a complex repertoire of secreted virulence factors [3,4,5,6]. Cell-free supernatants (secretomes) contain hundreds of bioactive molecules—including siderophores, capsular polysaccharides, toxins, outer membrane vesicles (OMVs), peptides, and metabolites—that modulate the host immune response, promote adhesion and invasion, and establish an immunosuppressive microenvironment [4,6,7,8]. For instance, *Enterobacter* spp. produce diverse exopolysaccharides and enzymes involved in stress adaptation and antibiotic resistance [9]. The secretome can suppress the oxidative burst in phagocytes, inhibit apoptosis of infected cells, and disrupt epithelial barrier integrity, thereby facilitating systemic dissemination of infection [10].

Analysis of such a chemically complex secretome remains methodologically challenging. Currently, mass spectrometry (MS) and nuclear magnetic resonance (NMR) spectroscopy are the primary platforms for metabolite profiling. MS offers high sensitivity and broad metabolite coverage but requires prior analyte separation (e.g., by chromatography) and is destructive, precluding sample reanalysis. In contrast, NMR spectroscopy does not require separation and preserves sample integrity; however, it suffers from significantly lower sensitivity—typically enabling identification of fewer than 100 metabolites in biological fluids—and demands larger sample volumes and more expensive instrumentation. Both techniques struggle with complex mixtures such as bacterial secretomes, where functionally relevant components may be present at low concentrations and masked by dominant signals [11].

Molecular methods such as PCR and next-generation sequencing (NGS) identify microorganisms based on nucleic acids but require extensive sample preparation, including removal of PCR inhibitors and DNA extraction. These approaches detect only genetic material, which may originate from dead cells or commensals, and do not reflect the actual secretion of bioactive molecules into the supernatant [12,13]. Moreover, NGS is costly, exhibits low sensitivity against background microbial “noise,” and often yields overly generalized taxonomic assignments [14].

In this context, Raman spectroscopy emerges as a promising alternative: it is non-invasive, non-destructive, compatible with aqueous environments (since water exhibits weak Raman scattering), requires minimal sample preparation, and provides a chemically informative “spectral fingerprint” in the native state [15,16]. Studies confirm that Raman spectra of bacterial supernatants contain characteristic signals corresponding to protein amide bonds, aromatic amino acids, nucleic acid phosphate backbones, and carbohydrates [17], enabling assessment of secretome composition without sample modification.

Despite its high information content, the application of Raman spectroscopy to complex biological mixtures such as bacterial secretomes is limited by spectral interference: signals from hundreds of components overlap, hindering unambiguous identification of individual molecules. To overcome this fundamental limitation, a hybrid approach combining chromatographic fractionation with subsequent Raman detection has been proposed. Preparative chromatography effectively reduces mixture complexity by separating components based on physicochemical properties (e.g., polarity, molecular weight), while Raman spectroscopy enables structural characterization of each fraction through its unique “spectral fingerprint” [18,19]. This strategy lowers the complexity of each fraction to an interpretable level, allowing distinctive peaks to serve as reliable indicators of potentially bioactive molecules involved in pathogenesis and intercellular communication. Critically, unlike mass spectrometry or nuclear magnetic resonance spectroscopy, this integrated workflow enables rapid, label-free, and non-destructive screening of functional metabolite fractions directly in biologically relevant aqueous media—offering a practical and scalable platform for secretome deconvolution in drug-resistant pathogens.

In this study, we applied the aforementioned combined approach to analyze the secretome of *Enterobacter* spp., a clinically relevant member of the ESKAPE group.

## 2. Results

After chromatographic fractionation of the cell-free supernatant of *Enterobacter* spp. into seven fractions and recording of Raman spectra in the range 300–2100 cm^−1^ for all seven fractions and the whole supernatant, the presence of both common and unique spectral features was revealed (Table 1) (Figure A2, Figure A3, Figure A4, Figure A5, Figure A6, Figure A7, Figure A8 and Figure A9).

Characteristic Raman bands at ~1682, 1470, 1282, 1080, 1040, and 870 cm^−1^ were consistently detected across all samples (fractions 1–7 and unfractionated supernatant), indicating core components of the *Enterobacter* spp. metabolic signature (Table 1).

Fraction 3 exhibited the most intense and complex spectral profile, characterized by the presence of several unique or significantly enhanced peaks compared to the other fractions and the unfractionated supernatant. Characteristic signals included (Table 1) (Figure A5): ~1682 cm^−1^, ~1546 cm^−1^, ~1470 cm^−1^, ~1446 cm^−1^, ~1342 cm^−1^, 1282 cm^−1^, ~1110 cm^−1^, ~1080 cm^−1^, ~1040 cm^−1^, ~1014 cm^−1^, ~980 cm^−1^, ~924 cm^−1^, ~850–820 cm^−1^, ~764 cm^−1^, and 464 cm^−1^.

The peak at 1682 cm^−1^ was present in all fractions; however, its intensity was maximal in fraction 3. Similarly, signals at 1282 cm^−1^, 1080 cm^−1^, and 1040 cm^−1^ were observed in all samples but reached their highest intensities in fraction 3. The peak at 1040 cm^−1^ was absent only in fraction 5. The peak at ~1446 cm^−1^, observed in fraction 3, exhibited significantly higher intensity compared to other fractions. It was only weakly detectable in fraction 4 and the unfractionated supernatant.

Unique peaks, either absent or present at very low intensity in other fractions, were registered in fraction 3 (Table 1): ~1546 cm^−1^, ~1446 cm^−1^, ~1342 cm^−1^, ~1214 cm^−1^, ~1110 cm^−1^, ~980 cm^−1^, ~924 cm^−1^, ~850 cm^−1^, ~830 cm^−1^, ~764 cm^−1^, ~728 cm^−1^, and 464 cm^−1^. The peak at ~980 cm^−1^ showed the highest intensity in fraction 3, was weakly expressed in fraction 2, and was practically absent in all other fractions. The peak at 464 cm^−1^ showed the highest intensity in fraction 3. However, a weak but discernible signal in this region was also detected in fraction 7, suggesting the possible presence of a shared component whose concentration varies across fractions.

A series of distinctive bands was observed in fractions other than fraction 3, indicating its unique composition. Notably, the band at ~1608 cm^−1^ was detected in all fractions except fraction 3.

In all fractions, except fractions 3, 5, 7, and the unfractionated supernatant, an intense peak at ~880 cm^−1^ was observed. This signal was absent or had significantly reduced intensity in these samples; instead, a peak at ~876 cm^−1^ was present, which was not detected in the other fractions. Likewise, peaks at 614 cm^−1^, 602 cm^−1^, 596 cm^−1^, 548 cm^−1^, and 498 cm^−1^ were found in all fractions except fractions 3 and 4. Particular attention should be paid to the spectral region between 682– and 692 cm^−1^. An intense peak at 686 cm^−1^ was observed in the spectrum of the unfractionated supernatant, but was absent in all fractions. Simultaneously, a peak at 688 cm^−1^ was observed in fractions 6 and 7, but not in fractions 3 and 4 (Figure A5 and Figure A6). A unique peak at ~930 cm^−1^ was present in the spectrum of the unfractionated supernatant but was not detected in all fractions. Conversely, a peak at ~924 cm^−1^ was observed exclusively in fraction 3.

Fraction 5 was characterized by the presence of two unique, intense peaks at ~1478 cm^−1^ and ~1488 cm^−1^, which were absent in the other fractions and the whole supernatant (Figure A7).

To correctly interpret the Raman spectra of the bacterial fractions, the spectrum of the original culture medium without bacteria was recorded. The medium contained Alpha-MEM (“Biolot”, St. Petersburg, Russia) supplemented with 0.2 mM myo-inositol, 0.02 mM folic acid, and 2 mM L-glutamine.

The following peaks (cm^−1^) were detected in the medium spectrum (Figure A10): ~1682 cm^−1^, ~1608 cm^−1^, ~1532 cm^−1^, ~1470 cm^−1^, ~1446 cm^−1^, ~1378 cm^−1^, ~1320 cm^−1^, ~1282 cm^−1^, ~1110 cm^−1^, ~1080 cm^−1^, ~1048 cm^−1^, ~1040 cm^−1^, ~1008 cm^−1^, ~982 cm^−1^, ~882 cm^−1^, ~834 cm^−1^, ~800 cm^−1^, ~770 cm^−1^, ~686 cm^−1^, ~602 cm^−1^, ~548 cm^−1^, and ~498 cm^−1^.

To simplify analysis and identify the origin of each signal, these peaks were compared with literature data on the Raman spectra of individual medium components. The comparison results are presented in four tables:

− Table 2: Peaks attributable to L-glutamine,
− Table 3: Peaks attributable to folic acid,
− Table 4: Peaks attributable to myo-inositol,
− Table 5: Peaks characteristic of Alpha MEM medium.

**Table 2:** Peaks Attributable to L-Glutamine.

Peak Position (Raman Shift, cm^−1^)	Group Assignment	References
~1682 cm^−1^	Amide I (C=O stretching in the peptide group of L-glutamine)	[32]
~1446 cm^−1^	CH_2_ scissor bending in L-glutamine	[32]
~1282 cm^−1^	Amide III (skeletal vibrations of C–N and N–H in peptide bonds; associated with tryptophan, tyrosine, histidine)	[23,32]
1050–1100 cm^−1^	C–C stretching in the skeleton of L-glutamine (ν(CC))	[25,32]
800 cm^−1^	CH_2_/NH_2_ deformations	[32]

**Table 3:** Peaks Attributable to Folic Acid.

Peak Position (Raman Shift, cm^−1^)	Group Assignment	References
1500–1650 cm^−1^	C=N and C=C stretching in the pteridine ring of folic acid	[33]
1378 cm^−1^	OH bending in pteridine and CH_2_ in the glutamate chain	[33]

Another component of the medium is myo-inositol—a cyclic polyol with six hydroxyl groups [34,35]. Although a complete attribution of Raman peaks to myo-inositol is not available in the literature, studies on structurally related polyols, particularly polytetramethylene glycol (PTMG), indicate its contribution manifests in the following peak regions: 1410–1487 cm^−1^, 1030–1120 cm^−1^, and 834–882 cm^−1^ (Table 4) [34,35].

**Table 4:** Peaks Attributable to Myo-Inositol.

Peak Position (Raman Shift, cm^−1^)	Group Assignment	References
~1682 cm^−1^	Amide I (C=O stretching in the peptide group of L-glutamine)	[32]
~1446 cm^−1^	CH_2_ scissor bending in L-glutamine	[32]
~1282 cm^−1^	Amide III (skeletal vibrations of C–N and N–H in peptide bonds; associated with tryptophan, tyrosine, histidine)	[23,32]
1050–1100 cm^−1^	C–C stretching in the skeleton of L-glutamine (ν(CC))	[25,32]
800 cm^−1^	CH_2_/NH_2_ deformations	[32]

According to the manufacturer’s instructions, Alpha MEM is an aqueous solution of purified inorganic salts, amino acids (including L-glutamine), vitamins, glucose, and phenol red indicator, sterilized by filtration through membrane filters with a pore size of no more than 0.22 μm. The medium contains no antibiotics and has a pH in the range of 7.1–7.5 [36]. The presence of a large number of substances in Alpha MEM complicates the precise identification of individual components within its composition.

Inorganic salts in aqueous solution do not yield intense Raman signals in the 300–2100 cm^−1^ range, as their ions lack complex vibrational modes. However, they can influence molecular conformation and hydrogen bonding, which indirectly alters the position and intensity of peaks from other components [37].

The vibrational modes of glucose (C_6_H_12_O_6_) are associated with valence vibrations of C–O, C–C, and C–H bonds. These signals are observed in the 800–1200 cm^−1^ range [38].

The peak at ~1446 cm^−1^ detected in the spectrum of the medium may be partially due to the presence of phenol red, an acid-base indicator included in Alpha MEM [39]. According to Sun et al. (2023), the characteristic peak of phenol red at 1441 cm^−1^ can likely undergo a slight shift up to 1446 cm^−1^ within a complex biological matrix, suggesting a potential contribution of the indicator to this signal [39].

Alpha MEM contains water-soluble vitamins and amino acids; however, the specific list of these compounds in the “Biolot” product is not provided by the manufacturer [36]. Due to the lack of precise data on the types and concentrations of individual substances, their direct attribution based on literature data is impossible. Consequently, assuming the contribution of specific substances to the registered spectrum of the original medium is incorrect.

Thus, chromatographic fractionation identified fraction 3 as the most enriched and spectrally unique, and also revealed several fraction-specific markers absent in both the whole unfractionated and the culture medium.

**Table 5:** Peaks Characteristic of Alpha MEM Medium.

Peak Position (Raman Shift, cm^−1^)	Group Assignment	References
~1342 cm^−1^	C–H deformation vibrations in protein structures	[23]
~1014 cm^−1^	C–C stretching in glutathione (glycine residue); possible glutathione-like peptides or sulfur-containing metabolites	[20]
~980 cm^−1^	Sulphate anions (SO_4_^2−^) and/or ammonium ions (NH_4_^+^); possible sulphated carbohydrates or exopolysaccharides	[27]
~924 cm^−1^	Unidentified band	-
~830 cm^−1^ and ~850 cm^−1^	Tyrosine Fermi doublet (symmetric ring breathing and overtone of bending; marker for tyrosyl residues)	[29,30]
~764 cm^−1^	Breathing vibration of the indole ring of tryptophan	[20,22,29]

## 3. Discussion

The obtained data demonstrate that the combination of chromatographic fractionation and Raman spectroscopy effectively resolves the complex secretome of *Enterobacter* spp. revealing distinct metabolite profiles across fractions. Of particular significance in this study is fraction 3, whose spectral profile markedly differs from the others, indicating enrichment with functionally significant biomolecules.

A key observation is the absence of the phenylalanine marker at ~1608 cm^−1^ in fraction 3, while it is present in all other fractions and the unfractionated supernatant [20]. According to Pezzotti (2021), the peak in this region (1607 cm^−1^) is associated with phenylalanine, specifically the C−O stretching vibration in its structure [20,21]. Furthermore, it has been established that the peak at 1607 cm^−1^ can partially overlap with the signal from tryptophan, which is typical for aromatic amino acids due to the similarity of their ring structures. The absence of this peak in fraction 3 indicates that this fraction is enriched with other classes of metabolites—for example, nucleic acids, polysaccharides, or specific peptides that do not contain phenylalanine in a detectable form. Thus, the peak at 1608 cm^−1^ serves as a marker for the presence of phenylalanine or phenylalanine-containing proteins/peptides in fractions 1, 2, 4, 5, 6, 7, and the whole cell-free supernatant. Its absence in fraction 3 underscores the uniqueness of its chemical composition [20].

Raman bands in the 1650–1700 cm^−1^ range can be associated with C=O vibrations characteristic of amide groups in proteins and peptides [19]. The high intensity of the peak at 1682 cm^−1^ exclusively in fraction 3 indicates enrichment with components rich in peptide or protein structures. This aligns with the overall profile of this fraction, which shows unique peaks in regions associated with nucleic acids (728, 1110 cm^−1^), carbohydrates (1040 cm^−1^), and aromatic amino acids. Thus, the enhancement of the signal at 1682 cm^−1^ in fraction 3 confirms the hypothesis that this fraction contains a complex metabolite composition, including peptides or protein fragments potentially involved in signaling processes, adhesion, or protection against oxidative stress.

In the spectrum of fraction 3, a pronounced peak is observed at ~1282 cm^−1^. This signal is present in other fractions as well, but its intensity is significantly lower in them. According to the literature, the peak at 1284 cm^−1^ corresponds to the amide III band (Amide III), which arises from skeletal vibrations of C–N and N–H bonds [23]. The presence of this signal suggests the enrichment of peptide structures containing aromatic or polar amino acid side chains, such as tryptophan, tyrosine, or histidine [23]. The high intensity of the peak at 1284 cm^−1^, observed exclusively in fraction 3, emphasizes its enrichment with peptides or proteins potentially involved in signaling processes, adhesion, or stress response. Its uniqueness highlights the specific composition of this fraction and requires further identification using LC-MS/MS methods.

This assumption is further supported by the presence of markers associated with of aromatic amino acids. Specifically, a pronounced and unique peak is observed at ~1546 cm^−1^ in Fraction 3, which is absent or has significantly lower intensity in the other fractions. According to Lee et al. (2021), the peak at 1546 cm^−1^, observed in the ordinary Raman spectrum (ORS) of the dipeptide L-alanyl-L-tryptophan (Ala-Trp) in the solid phase [22]. The authors unambiguously identified this band based on calculations within the framework of density functional theory (DFT) and comparison with known tryptophan spectra. In the same work, the amide-I band for Ala-Trp was clearly localized in the region of 1671–1685 cm^−1^ (depending on the aggregate state and pH) and was interpreted as the C=O stretching vibration combined with the N–H deformation (ν(C=O) + δ(NH)). This band is observed only in spectra obtained on citrate-reduced gold nanoparticles (CT-AuNPs) under acidic and neutral conditions, indicating the specific orientation of the peptide bond relative to the nanoparticle surface [22]. Thus, the presence of a pronounced peak at 1546 cm^−1^ in the spectra of the studied samples should be interpreted as a marker of the presence of the indole ring of tryptophan, rather than as a manifestation of the secondary structure of the peptide or protein. This is particularly important when analyzing complex biological mixtures, where intense bands of aromatic amino acids can be masked or mistakenly attributed to amide vibrations.

The peak at 764 cm^−1^, observed in fraction 3, is close to the characteristic band of 761–762 cm^−1^, which is unambiguously assigned in the literature to the symmetric ring-breathing mode (often referred to as ring breathing) of the indole ring in tryptophan. According to data presented in the review on Raman spectroscopy of functional amino acids, this frequency corresponds to the most intense band in the spectrum of free L-tryptophan and serves as a reliable marker for the presence of the indole fragment in biological samples [20]. In the study by Lee et al. (2021) on the dipeptide L-alanyl-L-tryptophan (Ala-Trp), a similar band was recorded at 750 cm^−1^ (in the solid phase) and 749 cm^−1^ (in an acidic aqueous medium) and also attributed to ν(R, r)—the breathing vibration of the indole ring [20,22,29]. The peak observed in our case at 764 cm^−1^ can be interpreted as a slightly shifted version of this marker band, which is typical for systems where tryptophan is part of a peptide chain or interacts with the microenvironment (e.g., the surface of nanoparticles, lipid structures, or ions) [20]. Such a shift of ~10–15 cm^−1^ is consistent with the known sensitivity of indole vibrations to conformation, hydrogen bonding, and the electrostatic environment [20]. The 762 cm^−1^ peak is also mentioned in the literature as a tryptophan marker in the context of differences from phenylalanine (whose main ring breathing band is located at 751 cm^−1^) [20,29]. Although the exact interpretation of the peak at 764 cm^−1^ is not provided in the literature, its unique presence in fraction 3 coincides with the detection of other signals associated with pyrimidine bases, such as the peak at 770 cm^−1^, associated with uracil [20]. Moreover, in a recent study by Zhang et al. (2025), modulation of regulators of pyrimidine nucleoside metabolism (e.g., PyrR) was shown to upregulate genes involved in de novo cytidine synthesis, resulting in the accumulation of cytidine [40]. Considering that fraction 3 exhibits the highest is characterized by maximum signal intensity and contains numerous peaks associated with nucleic acids and peptides, the peak at 764 cm^−1^ may be related to specific metabolic activity. This suggests that fraction 3 is a rich source of pyrimidine metabolites, potentially playing a role in intercellular communication or antibiotic resistance.

Furthermore, peaks at 830 cm^−1^ and 850 cm^−1^ should be noted, which are unique to fraction 3 and absent in the rest. These peaks form a characteristic Fermi doublet, typical for tyrosyl residues in proteins. As demonstrated in several studies, this doublet arises due to Fermi resonance between the symmetric ring breathing (ν_1_, A_1_ symmetry) and the overtone (2ν_16a_) of the out-of-plane ring bending in para-substituted benzenes. Both components of the doublet belong to the A_1_ symmetry and appear as strongly polarized bands [29,30]. A key feature of this doublet is that the intensity ratio of its components (I_850_/I_830_) is sensitive to the protonation state and hydrogen-bonding environment of the phenolic hydroxyl group [29,33]: (1) upon ionization of the phenolic group (pH > 11) or formation of a strong hydrogen bond where the hydroxyl hydrogen acts as a donor to a highly electronegative acceptor (e.g., carboxylate), the intensity of the band at 830 cm^−1^ exceeds the intensity of the band at 850 cm^−1^ (ratio I_850_/I_830_ ≈ 3:10–7:10); (2) in the case of weak or moderate hydrogen bonds, characteristic of “normal” (surface-exposed on the protein) tyrosyl residues, the band at 850 cm^−1^ predominates (ratio I_850_/I_830_ ≈ 10:4–10:8).

It is important to note that, as shown in the cited works, the intensity ratio of the doublet is practically independent of the conformation of the peptide backbone or the general hydrophobic environment of the aromatic ring, and is primarily determined by the electrostatic state of the phenolic oxygen [29,30,41].

Thus, the presence of both peaks—830 cm^−1^ and 850 cm^−1^—in the spectrum of fraction 3 unambiguously indicates the presence of tyrosine-containing structures, and their relative intensity can serve as a reliable indicator of the degree of ionization or the nature of hydrogen bonding of the phenolic group.

In all *Enterobacter* spp. fractions, except fractions 3, 5, 7, and the unfractionated supernatant, a pronounced peak is observed at ~880 cm^−1^. In these same samples, this signal is absent or has significantly reduced intensity; instead, a peak at ~876 cm^−1^ is present, which is not detected in the other fractions. According to Miura et al. (1988), peaks in the 870–880 cm^−1^ region relate to the vibrational modes of the indole ring of tryptophan (Trp) and correspond to its skeletal stretching (W17 band) [28]. The band position is sensitive to the Trp microenvironment [28]: at 880 cm^−1^—indicates a hydrophilic, solvent-exposed state; 876 cm^−1^ reflects a hydrophobic conformation within lipid or densely packed protein regions.

Thus, the spectral differences suggest that fractions 1, 2, 4, and 6 contain solvent-exposed tryptophan, indicative of soluble peptides, signaling molecules, or protein fragments, whereas fractions 3, 5, 7, and the unfractionated supernatant harbor tryptophan in a hydrophobic environment—consistent with localization in aggregates, extracellular vesicles, or membrane domains. This shift reflects distinct microenvironments across fractions and confirms the effectiveness of chromatographic separation in resolving components with differing structural organization and biological roles [28].

In fraction 3, a pronounced peak is observed at ~980 cm^−1^. This signal shows the highest intensity in fraction 3 and is also weakly expressed in fraction 2; it is practically absent in the other fractions. According to the literature, the peak at ~980 cm^−1^ may be associated with vibrations characteristic of sulphate anions (SO_4_^2−^) or ammonium derivatives (NH_4_^+^) in aqueous solutions [27]. This range is sensitive to the presence of ions involved in cellular metabolism, especially when analysing mixtures containing sulphates and ammonium salts [27]. However, the authors emphasize that this signal dominates in hetero-2D correlation spectra when studying ammonium sulphate solutions and correlates with SO_4_^2−^ bands in the IR spectrum [27].

Thus, the peak at ~980 cm^−1^, observed in the supernatant fractions of *Enterobacter* spp., may indicate the presence of sulphated compounds, such as sulphated carbohydrates, derivatives of sulphur-containing amino acids (e.g., cysteine), or exopolysaccharides modified with sulphate groups. Alternatively, it may be due to the presence of ammonium ions, which may be formed as a result of the degradation of nitrogen-containing metabolites.

In the spectrum of fraction 3 *Enterobacter* spp., a pronounced and unique peak is observed at ~1014 cm^−1^, which is absent or has significantly lower intensity in the other fractions. According to data from the work of Pezzotti (2021), this peak is associated with C–C stretching vibrations in the glutathione molecule—a tripeptide consisting of cysteine, glutamic acid, and glycine [20].

Thus, the peak at 1014 cm^−1^ can be described as a fingerprint of C–C stretching in the glycine residue (C_11_–C_12_) within the glutathione structure. Its presence indicates the possible presence of compounds containing a similar scaffold structure. At the time of writing the article, there is no data in the available scientific literature that unambiguously confirms the synthesis of glutathione by *Enterobacter* spp. However, the appearance of this signal may indicate the presence of: glutathione-like peptides having a similar C–C scaffold structure; protein fragments or metabolites whose conformations enhance the signal in this region; compounds modified with sulphur-containing groups whose vibrations overlap with the glutathione signal.

In the spectrum of fraction 3 *Enterobacter* spp., there is a pronounced peak at 464 cm^−1^, the intensity of which significantly exceeds that in other fractions. A weak but discernible signal in this region is also present in fraction 7, indicating the possible presence of a common component, the concentration of which, however, varies depending on the fraction. At the time of writing the article, there is no data in the available literature that allows unambiguous interpretation of the origin of the peak at 464 cm^−1^ in the context of bacterial metabolites or cellular components.

In the spectrum of fraction 3 *Enterobacter* spp., a pronounced and unique peak is observed at ~1214 cm^−1^, which is absent or has significantly lower intensity in the other fractions. At the time of writing the article, there is no data in the available scientific literature that allows unambiguous interpretation of the origin of this signal in the context of bacterial metabolites, proteins, nucleic acids, or lipids. The peak at 1214 cm^−1^ is not associated with any of the typical vibrational modes. Its appearance exclusively in fraction 3 indicates the presence of a specific component whose concentration reaches a detectable level only in this fraction [20]. The lack of data in the literature on this peak limit the possibility of its functional or chemical attribution. However, its uniqueness emphasizes the high degree of purification and separation of the supernatant components, which makes it a potential marker for differentiating fractions. To establish the nature of the substance responsible for the signal at 1214 cm^−1^, additional studies are needed, including mass spectrometry (LC-MS/MS) and comparison with model compounds.

Furthermore, a unique peak at ~1342 cm^−1^ is observed in fraction 3, which is absent in the other fractions. According to the literature and confirmed biochemical linkages [23], the peak in the 1342 cm^−1^ region is unambiguously associated with the deformation vibrations of C–H bonds in protein structures (CH deformation in proteins). This spectral marker is widely used as an indicator of the conformational state and protein content in biological samples, including stem cells, where its decrease correlates with oxidative damage to proteins and loss of viability [23]. In our case, conversely, the increased intensity of the 1342 cm^−1^ peak in fraction 3 indicates enrichment with protein or peptide components isolated from the supernatant. Considering that the analysis was carried out on acellular material, the source of the signal is probably secreted bacterial proteins, extracellular enzymes, toxins, or peptide signaling molecules (e.g., autoinducers or bacteriocins) that are stable under chromatographic separation conditions and retain characteristic vibrational modes of C–H bonds. The high intensity specifically in fraction 3 indicates the successful resolving power of the chromatographic system and suggests that this fraction contains functionally significant protein metabolites potentially responsible for the biological activity of the supernatant.

In the spectrum of fraction 3 *Enterobacter* spp., a pronounced peak is observed at ~1446 cm^−1^, the intensity of which significantly exceeds that in other fractions. A less intense, but discernible peak in this region is present in fraction 4 and in the whole cell-free supernatant, while in the other fractions it is absent or has minimal intensity. According to the literature [23], the peak at 1448 cm^−1^ (recorded as ~1446 cm^−1^ in our measurements, which corresponds to an acceptable spectral shift) corresponds to the deformation vibrations of C–H bonds (CH deformation) and is simultaneously present in DNA/RNA, proteins, lipids, and carbohydrates. This peak is an integral marker of the total content of biomacromolecules containing saturated hydrocarbon fragments, and is traditionally used as an internal standard for normalizing the Raman spectra of biological samples [23]. The selective enhancement of the signal at ~1446 cm^−1^ exclusively in fractions 3 and 4 indicates the concentration in these fractions of a mixture of biomolecules rich in C–H bonds—probably secreted proteins, nucleic acids, lipid components, or polysaccharides. The absence of the peak in the other fractions confirms the effectiveness of chromatographic separation and allows us to assume that fraction 3 contains the most concentrated set of C–H-containing metabolites of bacterial origin.

These data allow us to assume that fraction 3 contains a unique complex of metabolites, including peptides with tryptophan, but not containing phenylalanine-containing compounds. Its composition is significantly different from the other fractions and probably reflects the specific physiological activity or stress response of the bacteria.

Additionally, fraction 3 demonstrates pronounced signals characteristic of nucleic acids. In the spectrum of fraction 3 *Enterobacter* spp., a pronounced peak is observed at ~728 cm^−1^, which is present in other fractions with varying intensity. According to the literature, the peak at 728 cm^−1^ is associated with the ring deformation vibrations of nucleotides, in particular adenine—one of the purine bases of DNA and RNA [29]. This signal is a characteristic marker for nucleic acids and is often used in Raman spectroscopy to identify DNA and RNA in biological samples [29]. Its high intensity in fraction 3 indicates a significant accumulation of nucleic acid fragments—potentially fragments of DNA or RNA, free nucleotides, or adenine derivatives—in this fraction. The absence of this peak or its extremely low intensity in the other fractions emphasizes the uniqueness of the chemical composition of fraction 3 and may indicate that it is precisely in this fraction that the concentration or secretion of metabolites associated with nucleic acids occurs—for example, as a result of cell lysis, stress response, or active secretion of extracellular DNA (eDNA) involved in biofilm formation [29].

In the spectrum of fraction 3 *Enterobacter* spp., a pronounced and unique peak is observed at ~1110 cm^−1^, which is absent or has significantly lower intensity in the other fractions. This signal is in close proximity to the wavenumber of 1096 cm^−1^, which, according to data from the work of Okotrub et al. (2015), corresponds to the symmetric stretching vibrations of PO_2_^−^ bonds in the sugar-phosphate backbone of DNA [24]. The frequency shift from 1096 cm^−1^ to 1110 cm^−1^ may be due to changes in the conformation of DNA, its degree of hydration, ionic environment, or the presence of modifications. Such a shift is a known phenomenon in Raman spectroscopy and is often observed during transitions between the B- and Z-forms of DNA or upon protein binding. Moreover, according to Pezzotti (2021), peaks in the 1090–1100 cm^−1^ region can also overlap with signals from DNA and RNA, making them markers for differentiating bacterial strains [20,24].

Thus, the presence of a peak at 1110 cm^−1^ in fraction 3 indicates the possible presence of DNA fragments, potentially involved in the formation of extracellular networks (NETs) or intercellular communication [20,24]. Its uniqueness emphasizes the specific composition of this fraction and requires further verification using methods sensitive to nucleic acids, such as PCR or sequencing.

In the spectra of all *Enterobacter* spp. fractions, including the whole cell-free supernatant, a pronounced peak is observed at ~1080 cm^−1^. This signal shows the highest intensity in fraction 3, where it is one of the most dominant, while in the other fractions its intensity is significantly lower. The peak at ~1080 cm^−1^, according to data from Matthäus et al. (2008), corresponds to the symmetric stretching vibration of the phosphate group (ν_s_(PO_2_^−^)) in the phosphodiester bonds of nucleic acids and phospholipids [25]. In the cited work, this band is noted at 1083 cm^−1^ and unambiguously identified as O–P=O (symmetric stretching mode), characteristic of DNA, RNA, and phospholipids [25]. In the context of studying extracellular bacterial fractions, the presence of a pronounced peak in this region indicates the presence of phosphorus-containing biomolecules, such as extracellular DNA (eDNA), RNA, or components of membrane vesicles [20,25].

A substantial part of fraction 3’s profile consists of the signal at 1040 cm^−1^, which significantly exceeds the intensity of the analogous signal in other fractions. This peak is present, but with much lower intensity, in all fractions, except fraction 5, where it is absent. According to data from the work of Baron et al. (2020), the peak at 1040 cm^−1^ in the Raman spectra of bacteria is associated with vibrations characteristic of carbohydrates, including polysaccharides and other components of the extracellular matrix [26]. In their study, a significant increase in the signal in this region was observed, which the authors interpreted as a sign of the accumulation or redistribution of carbohydrate compounds [26]. This interpretation is consistent with data on the metabolism of *Enterobacter* spp., which are capable of utilizing a wide range of carbohydrates, including hexoses, pentoses, and their derivatives, through glycolysis, the pentose phosphate pathway, and the Entner–Doudoroff pathway [42]. The presence of these metabolic pathways makes it likely to produce polysaccharides, capsular components, or exopolysaccharides, especially under specific metabolic conditions.

Thus, the high intensity of the peak at 1040 cm^−1^ exclusively in fraction 3 indicates its enrichment with compounds containing C–O or C–C bonds, typical for polysaccharides, capsular components, or exopolysaccharides, which may be secreted into the supernatant. The absence of this signal in fraction 5 emphasizes the differences in the chemical composition between the fractions and indicates that this peak may serve as a marker for isolating fractions enriched with carbohydrate metabolites.

Additionally, peaks are observed in all fractions in the region close to ~870 cm^−1^ and ~1470 cm^−1^, which may be associated with CH_2_/CH_3_ group vibrations (deformation vibrations) and skeletal stretching in the hydrocarbon chains of lipids and proteins [20]. However, it is precisely the combination of the high intensity of the signal at 1080 cm^−1^ with other unique peaks in fraction 3—such as 980 cm^−1^, 1014 cm^−1^, and 1040 cm^−1^—that forms a specific “metabolic fingerprint,” sharply distinguishing this fraction from the others. This profile indicates that fraction 3 contains a complex set of metabolites, including not only carbohydrates, but also proteins, nucleic acids, and lipids, making it promising for further research in the context of virulence, biofilm formation, or intercellular signaling.

Of particular interest are the peaks at ~930 cm^−1^ and ~924 cm^−1^, demonstrating antagonistic behaviour: the peak at 930 cm^−1^ is observed only in the whole supernatant and completely disappears after chromatographic separation, while the peak at 924 cm^−1^ appears exclusively in fraction 3. This indicates that the original component responsible for the signal at 930 cm^−1^ is probably an aggregated or multi-component structure that breaks down during fractionation, while the 924 cm^−1^ peak reflects a high degree of purification of a specific metabolite. At the time of writing the article, there is no data in the literature that allows unambiguous interpretation of the origin of these signals. Their nature may be related to previously unidentified metabolites, post-translational modifications, or specific environmental conditions, and requires verification by LC-MS/MS or NMR spectroscopy methods.

Additionally, a pronounced peak at 496 cm^−1^ is observed in all fractions, except fractions 3 and 4. In fractions 3 and 4, this peak is present, but with significantly lower intensity. At the time of writing the article, there is no data in the available literature that allows unambiguous interpretation of the origin of the peak at 496 cm^−1^ in the context of bacterial metabolites or cellular components. Its presence in most fractions indicates a possible connection with common components of the supernatant; however, its absence or weak intensity in fractions 3 and 4 emphasizes the uniqueness of the chemical composition of these fractions and requires further study.

A key observation is the presence of a peak at 548 cm^−1^ in all fractions, except fractions 3 and 4. According to Pezzotti (2021), the peak at 548 cm^−1^ corresponds to the deformation vibrations of methylene groups (CH_2_) in the cholesterol ring (CHL) and is one of its characteristic fingerprints in the Raman spectrum [20]. Although cholesterol is not a component of the bacterial cell wall, its spectral features can be reproduced by structurally similar molecules—for example, cyclic sterol-like lipids, isoprenoids, or modified terpenoids that may be secreted by bacteria into the supernatant [31]. The absence of this peak in fractions 3 and 4 indicates that these fractions are enriched with other classes of metabolites—proteins, nucleic acids, and polysaccharides—and do not contain lipids that give a signal at 548 cm^−1^. This is consistent with data on the phospholipid composition of bacterial membranes, where the main components are phosphatidylethanolamine (PE), phosphatidylglycerol (PG), and cardiolipin (CL), and not sterols [31].

The presence of the 548 cm^−1^ peak in fractions 1, 2, 5, 6, and 7 may indicate the presence of cyclic lipids involved in the formation of extracellular vesicles, membrane domains, or signaling molecules, which requires further identification by LC-MS methods.

Weak but discernible peaks at 596 cm^−1^, 602 cm^−1^, and 614 cm^−1^ are observed in all fractions, except fractions 3 and 4. These signals are absent or practically not expressed in fractions 3 and 4, which further emphasizes their difference in chemical composition. At the time of writing the article, there is no data in the available literature that allows unambiguous interpretation of the origin of these peaks in the context of bacterial metabolites or cellular components. Their presence in most fractions may indicate the presence of common structural elements, possibly related to the deformation vibrations of cyclic systems, C–C bonds in rigid conformations, or vibrations in the composition of lipid or polysaccharide backbones [20]. However, without additional data on similar systems, their nature remains unclear.

Special attention is warranted for the spectral profile in the 682–692 cm^−1^ region. In the spectrum of the whole supernatant, a pronounced peak is observed at 686 cm^−1^, absent in all fractions. Simultaneously, a peak at 688 cm^−1^ is fixed in fractions 6 and 7, which is absent in fractions 3 and 4. According to Pezzotti (2021), the 682–692 cm^−1^ range corresponds to C–S stretching in methionine and its dimer, indicating the presence of sulphur-containing amino acids or their derivatives involved in sulfur metabolism and biofilm formation [20]. The presence of the 686 cm^−1^ peak only in the whole supernatant may indicate that this compound represents an unstable or easily degradable metabolite that breaks down or redistributes during chromatographic separation. The appearance of the 688 cm^−1^ peak in fractions 6 and 7 indicates a partial preservation of sulphur-containing components in these fractions, while their complete absence in fractions 3 and 4 confirms the radical difference in the chemical composition of these fractions—probably due to enrichment with protein, nucleic acid, and polysaccharide components that do not contain sulphur.

In the spectrum of fraction 5 *Enterobacter* spp., the presence of two pronounced and unique peaks at ~1478 cm^−1^ and ~1488 cm^−1^ is observed, which are absent or have minimal intensity in the other fractions and in the cell-free supernatant. These signals are key markers that distinguish fraction 5 from all others. According to data from the work of Pezzotti (2021) [20], peaks in the 1476 cm^−1^ and 1486 cm^−1^ regions are associated with vibrations characteristic of nucleic acids. In particular: the peak at 1476 cm^−1^ corresponds to C–H deformation and CH_3_ bending in the structure of pyrimidine bases, such as deoxythymidine triphosphate (dTTP), and the peak at 1486 cm^−1^ relates to ring vibrations in the structure of DNA/RNA, including C–N stretching in adenine and cytosine. This signal (1486 cm^−1^) was identified as one of the diagnostic markers for differentiating Gram-negative and Gram-positive bacteria [20].

Thus, the observed peaks at 1478 cm^−1^ and 1488 cm^−1^ in fraction 5 can be interpreted as manifestations of structural features of nucleic acids—potentially, fragments of DNA or RNA with a high content of thymine (T) and adenine (A). Their uniqueness indicates that it is precisely in this fraction that the concentration of nucleic acid metabolites occurs. This may be related to processes such as cell lysis, active secretion of extracellular DNA (eDNA) involved in biofilm formation, or the accumulation of free nucleotides under stationary growth phase conditions.

It should be noted that the exact attribution of signals in the spectra of fractions is difficult due to the complex composition of the original culture medium (α-MEM), which contains many components, each of which can give its own Raman signals. Comparing the spectra of the whole supernatant and individual fractions with the background spectrum of pure medium allows the isolation of signals likely related to the products of bacterial life. Based on this analysis, the following conclusions can be drawn: (1) Peaks present in the medium and in all samples (supernatant, all fractions): ~1682 cm^−1^, ~1470 cm^−1^, ~1282 cm^−1^, ~1080 cm^−1^. For these signals, it is impossible to determine their origin exactly: they can be due to both medium components and bacterial products; (2) Peaks characteristic of the medium and not found in some fractions: Peak at ~1608 cm^−1^, ~1532 cm^−1^, ~1446 cm^−1^, ~1378 cm^−1^, ~1320 cm^−1^, ~1110 cm^−1^, ~1048 cm^−1^, ~1040 cm^−1^, ~1008 cm^−1^, ~1008 cm^−1^, ~982 cm^−1^, ~800 cm^−1^, ~770 cm^−1^, ~686 cm^−1^, ~602 cm^−1^, ~548 cm^−1^. These signals are most likely markers of medium components that are masked or degraded during chromatographic separation. (3) Peaks specific to bacterial products (absent in the medium): Signals that are absent in the spectrum of pure medium but present in the supernatant and/or fractions can most likely be identified as products of bacterial metabolism. These include: ~1546 cm^−1^, ~1488 cm^−1^, ~1478 cm^−1^, ~1342 cm^−1^, ~1214 cm^−1^, ~1014 cm^−1^, ~980 cm^−1^, ~930 cm^−1^, ~880 cm^−1^, ~876 cm^−1^, ~870 cm^−1^, ~830 cm^−1^, ~764 cm^−1^, ~728 cm^−1^, ~688 cm^−1^, ~614 cm^−1^, ~596 cm^−1^, ~496 cm^−1^, ~464 cm^−1^.

Thus, the combined approach of chromatographic fractionation and Raman spectroscopy successfully allowed the deconstruction of the complex secretome of the Gram-negative pathogen *Enterobacter* spp., overcoming the problem of spectral overload. A key finding was the identification of fraction 3 as unique in its metabolite profile: it is characterized by the absence of phenylalanine markers (~1608 cm^−1^) and sterol-like lipids (548 cm^−1^), sharply distinguishing it from the other fractions and the whole supernatant. This fraction demonstrates maximum signal intensity indicating enrichment with functionally significant biomolecules—in particular, peptides containing tryptophan and tyrosine (peaks ~1546, ~764, ~830–850 cm^−1^), fragments of nucleic acids (peaks ~1080, ~1110, ~728 cm^−1^), and polysaccharides (peak ~1040 cm^−1^). The presence of a signal at ~1014 cm^−1^ may indicate the presence of glutathione-like compounds, emphasizing the potential role of this fraction in protection against oxidative stress. The combination of these specific signals suggests that fraction 3 represents a functionally specialized part of the secretome, potentially carrying key virulence or signaling functions, such as adhesion, immunosuppression, and biofilm formation. The obtained data confirm that chromatography effectively reduces spectral complexity, allowing Raman spectroscopy to identify unique “metabolic fingerprints” of individual fractions. Additional studies using mass spectrometry and molecular biology methods are needed for the final identification of the components of fraction 3. Overall, the proposed method is a promising strategy for deep metabolomic profiling of bacterial secretomes and can be applied to identify new targets for the diagnosis and therapy of infections caused by antibiotic-resistant pathogens.

## 4. Materials and Methods

### 4.1. Cell Cultures

The *Enterobacter* spp. strain (ATCC 13047) was used in this study. Bacteria were cultured on agarose medium under appropriate biosafety containment, in accordance with institutional safety protocols for handling pathogenic microorganisms.

### 4.2. Preparation of Conditioned Media Following the Cultivation of ESKAPE Bacteria

To prepare conditioned media, bacterial cultures were inoculated into a medium containing α-MEM (Biolot, St. Petersburg, Russia), 0.2 mM myo-inositol, 0.02 mM folic acid, and 2 mM L-glutamine to a final concentration of 1 × 10^8^ CFU/mL. Cultivation was performed in sterile glass vials containing 5 mL of growth medium for 24 h at 37 °C. After the incubation, the supernatant was filtered through 0.22 μm pore-size syringe filters (Sarstedt, Nümbrecht, Germany) to ensure sterility purity.

### 4.3. Preparative Chromatography

**Figure A1:**
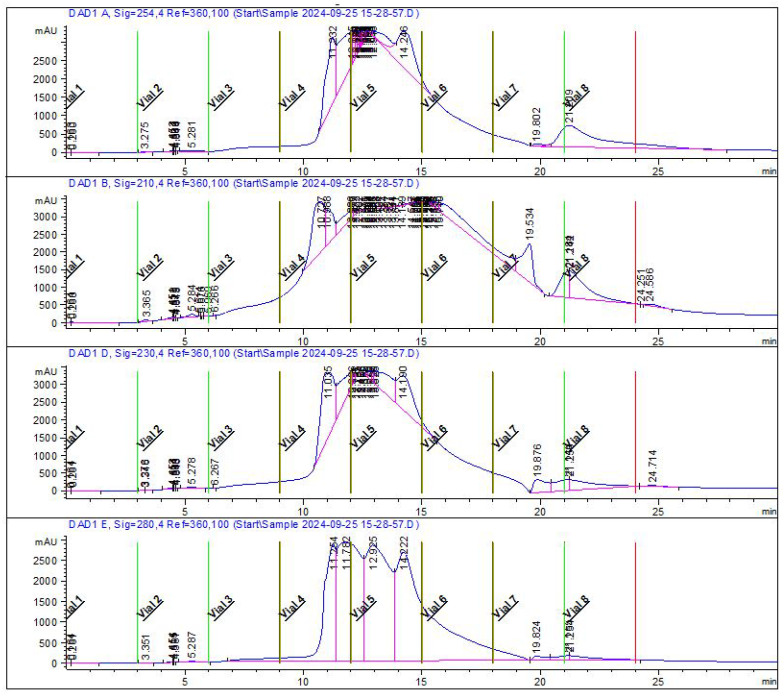
DAD detector chromatograms at wavelengths of 254.4 nm (A), 210.4 nm (B), 230.4 nm (D), and 280.4 nm (E), obtained during preparative separation of protein fractions from bacterial supernatants by gel filtration on a Bio SEC-3 column (Agilent). Peak retention times are indicated above the corresponding peaks. Samples: Vial 1—concentrated input supernatant; Vials 2–8—sequential fractions collected in time-line mode at 3-min intervals (Fractions 1–7, respectively). Analysis was performed under isocratic conditions at 4 °C, with a flow rate of 0.35 mL/min and distilled water as the mobile phase. Fractions collected after 24 min contained no detectable protein and were not further analyzed.

**Figure A2:**
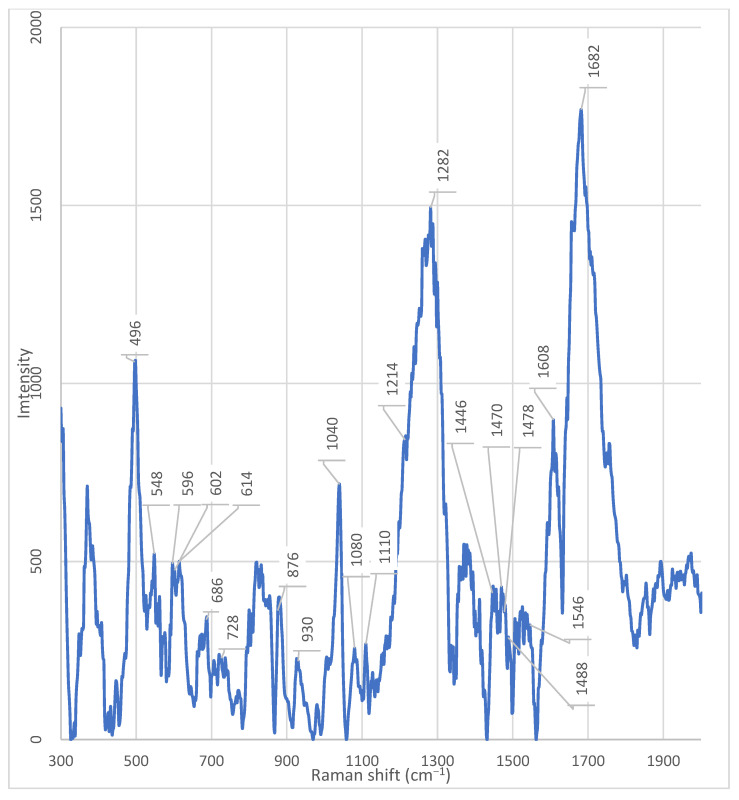
Raman spectrum of the whole cell-free supernatant of *Enterobacter* spp. in the range of 300–2100 cm^−1^.

**Figure A3:**
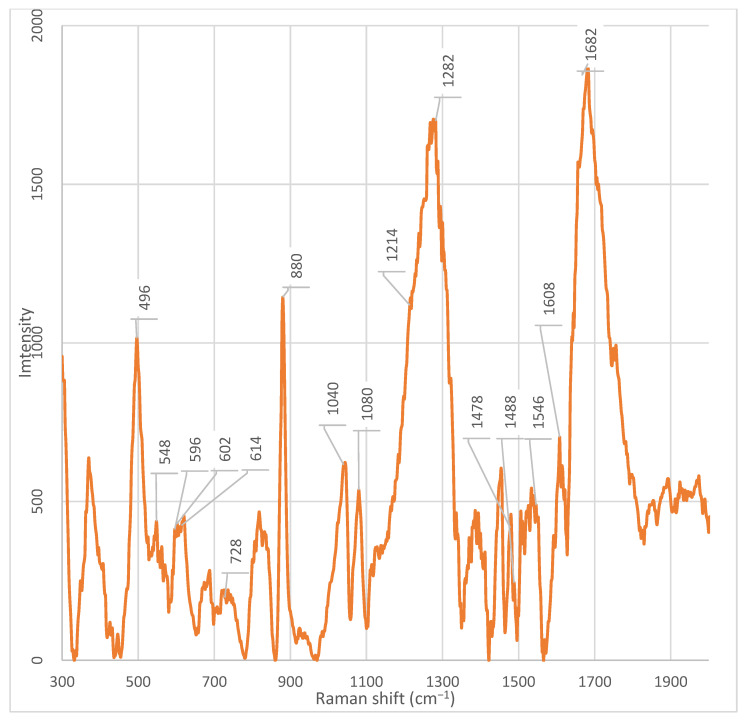
Raman spectrum of fraction 1 of *Enterobacter* spp. in the range of 300–2100 cm^−1^.

**Figure A4:**
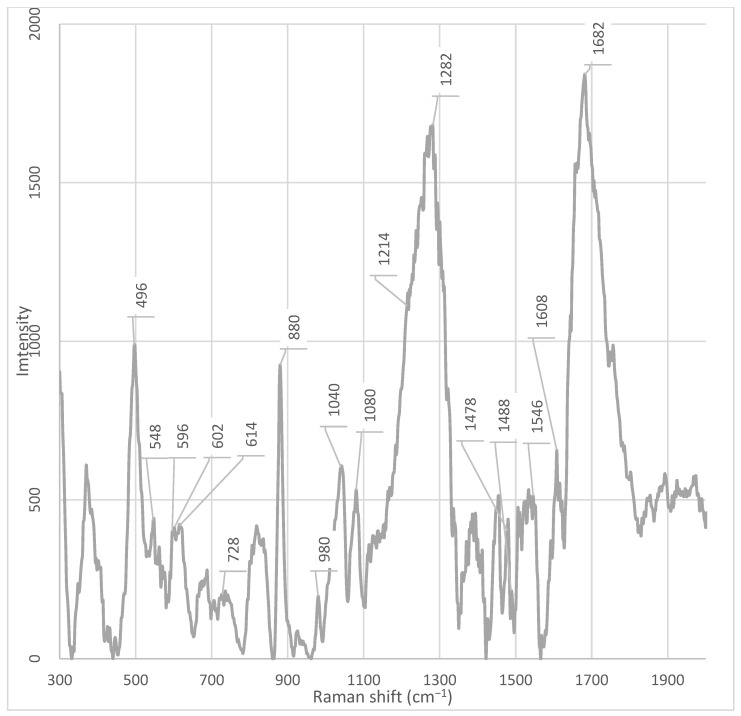
Raman spectrum of fraction 2 of *Enterobacter* spp. in the range of 300–2100 cm^−1^.

**Figure A5:**
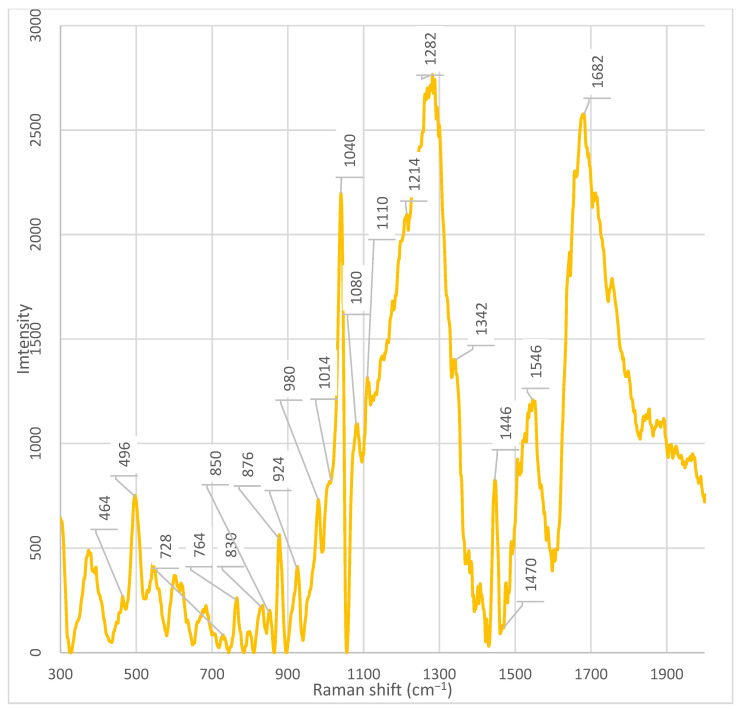
Raman spectrum of fraction 3 of *Enterobacter* spp. in the range of 300–2100 cm^−1^.

**Figure A6:**
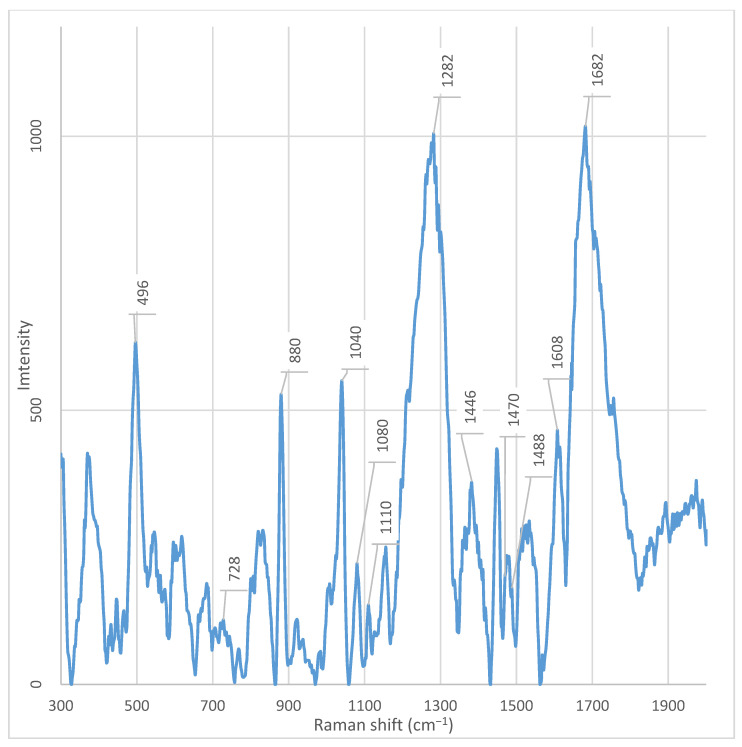
Raman spectrum of fraction 4 of *Enterobacter* spp. in the range of 300–2100 cm^−1^.

**Figure A7:**
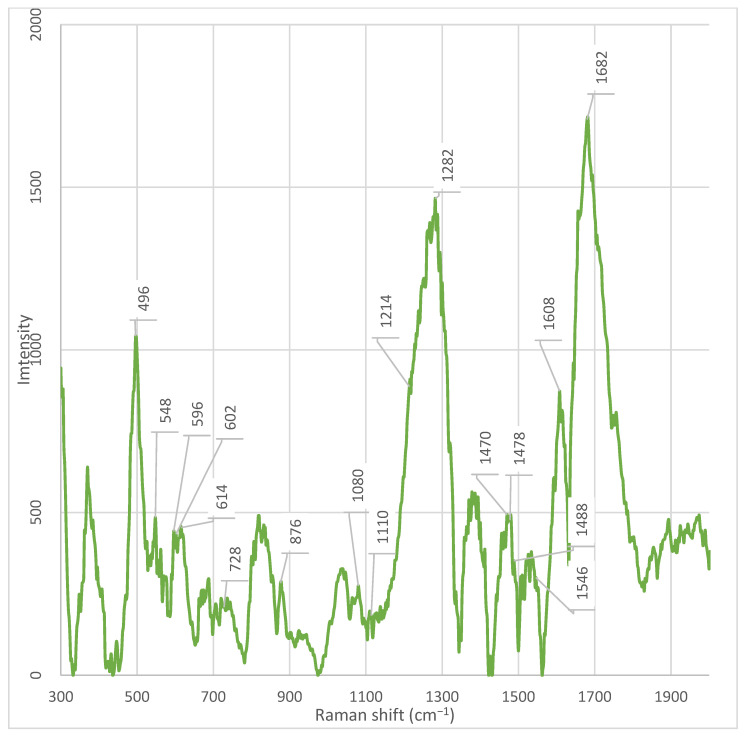
Raman spectrum of fraction 5 of *Enterobacter* spp. in the range of 300–2100 cm^−1^.

**Figure A8:**
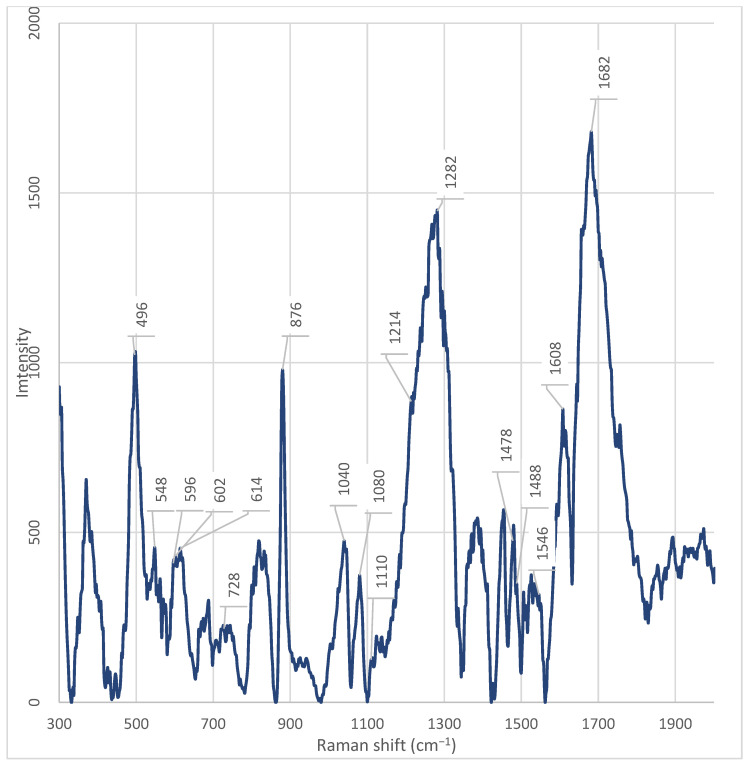
Raman spectrum of fraction 6 of *Enterobacter* spp. in the range of 300–2100 cm^−1^.

**Figure A9:**
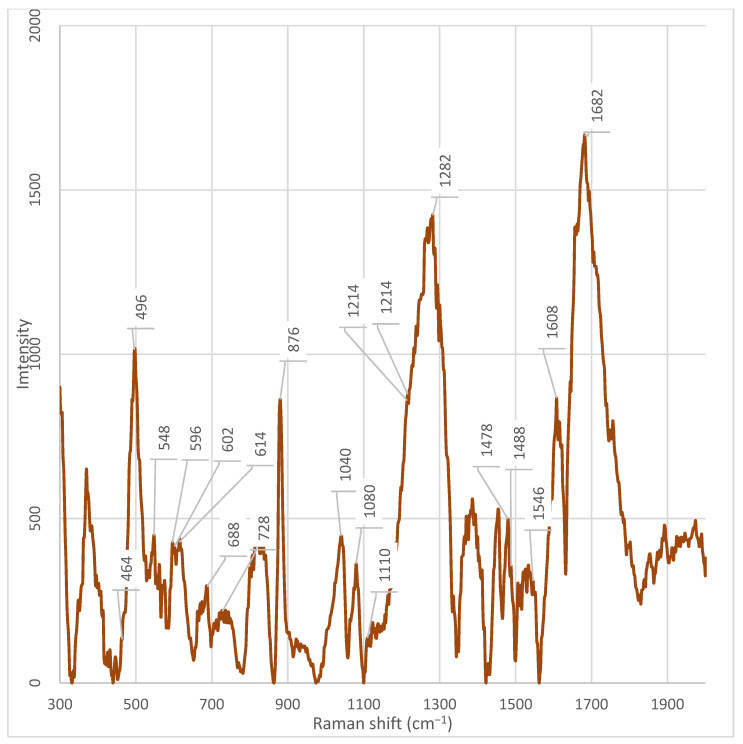
Raman spectrum of fraction 7 of *Enterobacter* spp. in the range of 300–2100 cm^−1^.

**Figure A10:**
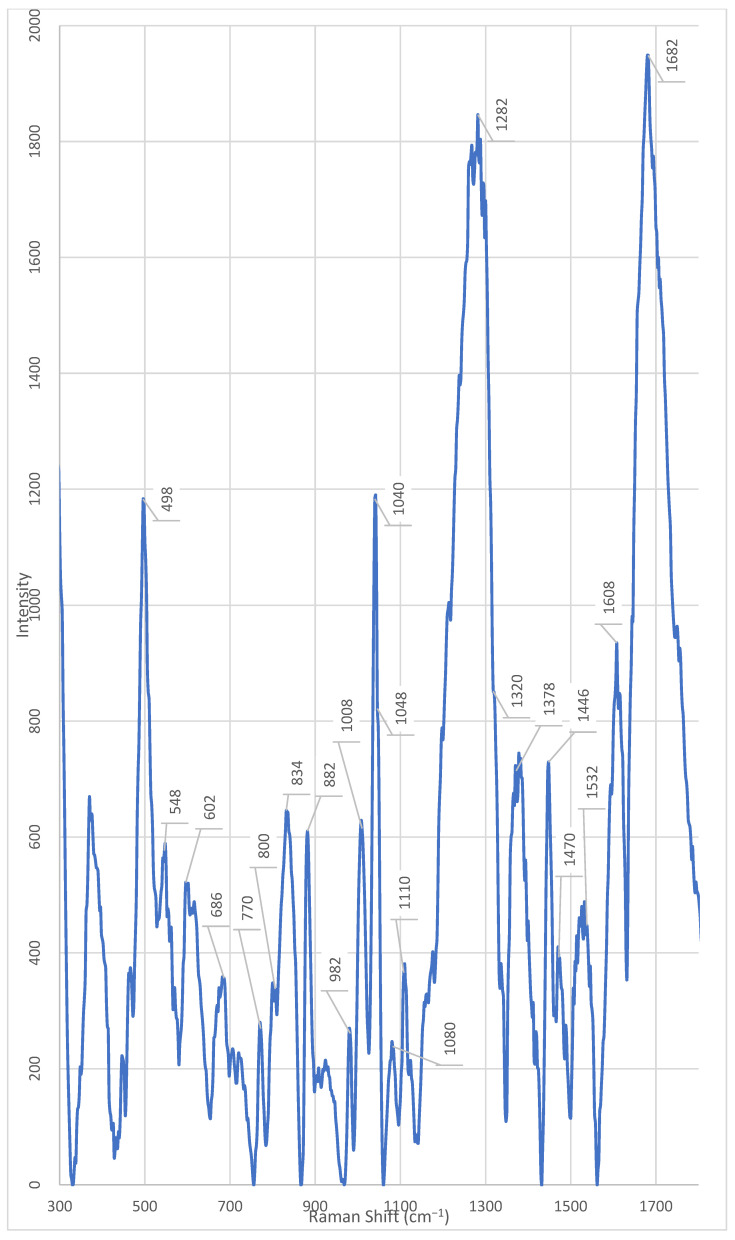
Raman spectrum of the culture medium for *Enterobacter* spp. prior to inoculation (300–2100 cm^−1^).

Bacterial cell-free supernatants were concentrated using a CentriVap Vacuum Concentrator (Labconco Corp., Kansas City, MO, USA) to a volume where the total protein concentration exceeded the threshold of 5 mg/mL. To separate the concentrated supernatants into protein fractions, a high-performance liquid chromatograph 1260 Infinity II (Agilent Technologies, Inc., Santa Clara, CA, USA) equipped with OpenLAB CDS ChemStation software (version C.XX.XX, Agilent Technologies, Inc., USA) was used (Figure A1). Chromatographic separation was conducted under non-denaturing conditions on an Agilent Bio SEC-3 Size Exclusion Column, 3 μm, 300 Å, 4.6 × 300 mm (Agilent Technologies, Inc., USA). Deionized water was used as the mobile phase. Analysis was performed in isocratic mode at +4 °C with a mobile phase flow rate of 0.35 mL/min. A diode array detector was used to record absorbance at 210, 230, 254, and 280 nm. The analysis duration was 30 min. Due to the continuous nature of the chromatographic profile; fractionation was performed in time-line mode over 3-min intervals, starting from the 3rd min (from the 24th min after elution from the chromatographic column and subsequent protein concentration, no protein was detected, which allowed exclusion of this material from further work). The obtained fractions were concentrated using a CentriVap Vacuum Concentrator to a volume of 2.5 mL—this volume was selected to standardize the protocol, as total protein levels were below the detection limit. The samples were then sterilized by filtration through 0.22 μm pore-size syringe filters (Sarstedt, Nümbrecht, Germany), frozen at −80 °C, and stored for up to two weeks prior to analysis.

### 4.4. Raman Spectroscopy

Raman spectra were recorded using a 785-nm laser (300 mW) on an OPTEC 785LRam spectrometer (OPTEC, Moscow, Russia). The spectral range was 300–2100 cm^−1^, with a spectral resolution of 8 cm^−1^. Samples were analyzed in liquid phase in 2 cm^3^ glass vials. Due to the strong fluorescence background, spectral analysis was performed using the second derivative of spectra acquired with 785-nm excitation. Prior to spectral acquisition, samples were brought to room temperature and thoroughly mixed. Baseline correction was performed using iterative polynomial approximation. Data processing was carried out using algorithms implemented in the BWSpec4 and OPTEC-Raman version 1.2 software packages. Due to the strong fluorescence background, spectral analysis was performed using the second derivative of spectra acquired with 785-nm excitation. This allowed compensation for the fluorescent background, identification of often hidden peaks, and detection of differences in spectral band parameters such as wavenumber position, bandwidth at half maximum height, and area under the spectral contour.

## 5. Conclusions

In this study, we developed and applied a combined approach integrating chromatographic fractionation and Raman spectroscopy to resolve the complex secretome of the Gram-negative ESKAPE pathogen *Enterobacter* spp. We demonstrated that this method effectively overcomes the problem of spectral congestion and enables the identification of functionally significant fractions inaccessible to analysis in the whole supernatant.

Of particular interest is fraction 3, distinguished by a unique metabolite profile: it is enriched with peptides containing tryptophan and tyrosine, fragments of nucleic acids, polysaccharides, and potentially glutathione-like compounds, while completely lacking markers of phenylalanine and sterol-like lipids. Such a composition suggests a potential role for this fraction in key pathogenic processes—adhesion, biofilm formation, intercellular communication, and modulation of the immune response.

The proposed approach opens new possibilities for the targeted isolation and identification of bioactive molecules involved in pathogen-host interactions.

Therefore, the combination of chromatography and Raman spectroscopy represents a promising strategy for in-depth metabolomic profiling of bacterial secretomes and can be utilized to identify novel diagnostic and therapeutic targets for infections caused by antibiotic-resistant pathogens.

## Figures and Tables

**Table 1:** Matching of Raman peaks detected in this study to molecular structures.

Peak Position (Raman Shift, cm^−1^)	Group Assignment	Detection	References
~1608 cm^−1^	C-C stretching in the aromatic ring of phenylalanine (partially overlaps with tryptophan)	Whole supernatant, fractions 1, 2, 4, 5, 6, 7	[20,21]
~1546 cm^−1^	Indole ring of tryptophan	Whole supernatant, fractions 1, 2, 3, 5, 6, 7	[22]
~1488 cm^−1^	Ring vibrations and C–N stretching in purine and pyrimidine bases (adenine, guanine, cytosine)	Whole supernatant, fractions 1, 2, 4, 5, 6, 7	[20]
~1478 cm^−1^	C–H deformation and CH_3_ bending in pyrimidine bases (e.g., thymine)	Whole supernatant, fractions 1, 2, 5, 6, 7	[20]
~1470 cm^−1^ and ~870 cm^−1^	Deformational and skeletal vibrations of CH_2_/CH_3_ in hydrocarbon chains of lipids and proteins	Whole supernatant, fractions 1, 2, 3, 4, 5, 6, 7	[20]
~1446 cm^−1^	C–H deformational vibrations (proteins, lipids, nucleic acids, carbohydrates)	Whole supernatant, fractions 3, 4	[23]
~1342 cm^−1^	C–H deformational vibrations in protein structures	Fraction 3	[23]
~1282 cm^−1^	Amide III (skeletal vibrations of C–N and N–H in peptide bonds; associated with Trp, Tyr, His)	Whole supernatant, fractions 1, 2, 3, 4, 5, 6, 7	[23]
~1214 cm^−1^	Unidentified band	Whole supernatant, fractions 1, 2, 3, 5, 6, 7	-
~1110 cm^−1^	Sugar-phosphate backbone of DNA (symmetric PO_2_^−^ stretching); possible conformational changes or modifications	Whole supernatant, fractions 3, 4, 5, 6, 7	[19,24]
~1080 cm^−1^	Symmetric phosphate group stretching ν_s_(PO_2_^−^) in nucleic acids and phospholipids (O–P=O)	Whole supernatant, fractions 1, 2, 3, 4, 5, 6, 7	[25]
~1040 cm^−1^	Carbohydrates (polysaccharides, exopolysaccharides, components of the extracellular matrix)	Whole supernatant, fractions 1, 2, 3, 4, 6, 7	[26]
~1014 cm^−1^	C–C stretching in glutathione (glycine residue); possible glutathione-like peptides or sulfur-containing metabolites	Fraction 3	[20]
~980 cm^−1^	Sulphate anions (SO_4_^2−^) and/or ammonium ions (NH_4_^+^); possible sulphated carbohydrates or exopolysaccharides	Fractions 2, 3	[27]
~930 cm^−1^	Unidentified band	Whole supernatant	-
~924 cm^−1^	Unidentified band	Fraction 3	-
~880 cm^−1^	Skeletal stretching of the indole ring of tryptophan (W17) in a hydrophilic (aqueous) environment	Fractions 1, 2, 4, 6	[28]
~876 cm^−1^	Skeletal stretching of the indole ring of tryptophan (W17) in a hydrophobic environment	Whole supernatant, fractions 3, 5, 7	[28]
~830 cm^−1^ and ~850 cm^−1^	Tyrosine Fermi doublet (symmetric ring breathing and overtone of bending; marker for tyrosyl residues)	Fraction 3	[29,30]
~764 cm^−1^	Breathing vibration of the indole ring of tryptophan	Fraction 3	[20,22,29]
~728 cm^−1^	Ring deformational vibrations of adenine (purine base of DNA/RNA)	Whole supernatant, fractions 1, 2, 3, 4, 5, 6, 7	[29]
~686–688 cm^−1^	C–S stretching in methionine and its dimer (sulfur-containing amino acids)	Whole supernatant, fractions 6, 7	[20]
~614 cm^−1^	Unidentified band	Whole supernatant, fractions 1, 2, 5, 6, 7	-
~602 cm^−1^	Unidentified band	Whole supernatant, fractions 1, 2, 5, 6, 7	-
~596 cm^−1^	Unidentified band	Whole supernatant, fractions 1, 2, 5, 6, 7	-
~548 cm^−1^	CH_2_ deformational vibrations in sterol-like lipids (cholesterol-like structures, isoprenoids, terpenoids)	Whole supernatant, fractions 1, 2, 5, 6, 7	[20,31]
~496 cm^−1^	Unidentified band	Whole supernatant, fractions 1, 2, 3, 4, 5, 6, 7	-
~464 cm^−1^	Unidentified band	Fractions 3, 7	-
~1040 cm^−1^	Carbohydrates (polysaccharides, exopolysaccharides, components of the extracellular matrix)	Whole supernatant, fractions 1, 2, 3, 4, 6, 7	[26]
~1014 cm^−1^	C–C stretching in glutathione (glycine residue); possible glutathione-like peptides or sulfur-containing metabolites	Fraction 3	[20]
~980 cm^−1^	Sulphate anions (SO_4_^2−^) and/or ammonium ions (NH_4_^+^); possible sulphated carbohydrates or exopolysaccharides	Fractions 2, 3	[27]
~930 cm^−1^	Unidentified band	Whole supernatant	-
~924 cm^−1^	Unidentified band	Fraction 3	-
~880 cm^−1^	Skeletal stretching of the indole ring of tryptophan (W17) in a hydrophilic (aqueous) environment	Fractions 1, 2, 4, 6	[28]
~876 cm^−1^	Skeletal stretching of the indole ring of tryptophan (W17) in a hydrophobic environment	Whole supernatant, fractions 3, 5, 7	[28]
~830 cm^−1^ and ~850 cm^−1^	Tyrosine Fermi doublet (symmetric ring breathing and overtone of bending; marker for tyrosyl residues)	Fraction 3	[29,30]
~764 cm^−1^	Breathing vibration of the indole ring of tryptophan	Fraction 3	[20,22,29]
~728 cm^−1^	Ring deformational vibrations of adenine (purine base of DNA/RNA)	Whole supernatant, fractions 1, 2, 3, 4, 5, 6, 7	[29]
~686–688 cm^−1^	C–S stretching in methionine and its dimer (sulfur-containing amino acids)	Whole supernatant, fractions 6, 7	[20]
~614 cm^−1^	Unidentified band	Whole supernatant, fractions 1, 2, 5, 6, 7	-
~602 cm^−1^	Unidentified band	Whole supernatant, fractions 1, 2, 5, 6, 7	-
~596 cm^−1^	Unidentified band	Whole supernatant, fractions 1, 2, 5, 6, 7	-
~548 cm^−1^	CH_2_ deformational vibrations in sterol-like lipids (cholesterol-like structures, isoprenoids, terpenoids)	Whole supernatant, fractions 1, 2, 5, 6, 7	[20,31]
~496 cm^−1^	Unidentified band	Whole supernatant, fractions 1, 2, 3, 4, 5, 6, 7	-
~464 cm^−1^	Unidentified band	Fractions 3, 7	-

## Data Availability

The original contributions presented in this study are included in the article. Further inquiries can be directed to the corresponding author.

## References

[1] Pendleton J.N., Gorman S.P., Gilmore B.F. (2013). Clinical relevance of the ESKAPE pathogens. Expert Rev. Anti Infect. Ther..

[2] World Health Organization (2024). WHO Bacterial Priority Pathogens List, 2024: Bacterial Pathogens of Public Health Importance to Guide Research, Development and Strategies to Prevent and Control Antimicrobial Resistance.

[3] Davin-Regli A., Pagès J.M. (2015). *Enterobacter aerogenes* and *Enterobacter cloacae*; versatile bacterial pathogens confronting antibiotic treatment. Front. Microbiol..

[4] Busatto S., Zendrini A., Radeghieri A., Paolini L., Romano M., Presta M., Bergese P. (2019). The Nanostructured Secretome. Biomater. Sci..

[5] March C., Cano V., Moranta D., Llobet E., Pérez-Gutiérrez C., Tomás J.M., Suárez T., Garmendia J., Bengoechea J.A. (2013). Role of bacterial surface structures on the interaction of *Klebsiella pneumoniae* with phagocytes. PLoS ONE.

[6] Uppalapati S.R., Sett A., Pathania R. (2020). The Outer Membrane Proteins OmpA, CarO, and OprD of Acinetobacter baumannii Confer a Two-Pronged Defense in Facilitating Its Success as a Potent Human Pathogen. Front. Microbiol..

[7] Aperce C.C., Burkey T.E., KuKanich B., Crozier-Dodson B.A., Dritz S.S., Minton J.E. (2010). Interaction of *Bacillus* species and Salmonella enterica serovar Typhimurium in immune or inflammatory signaling from swine intestinal epithelial cells. J. Anim. Sci..

[8] Thiel M., Caldwell C.C., Sitkovsky M.V. (2003). The critical role of adenosine A2A receptors in downregulation of inflammation and immunity in the pathogenesis of infectious diseases. Microbes Infect..

[9] Denissen J., Reyneke B., Waso-Reyneke M., Havenga B., Barnard T., Khan S., Khan W. (2022). Prevalence of ESKAPE pathogens in the environment: Antibiotic resistance status, community-acquired infection and risk to human health. Int. J. Hyg. Environ. Health.

[10] Daca A., Jarzembowski T. (2024). From the Friend to the Foe-*Enterococcus faecalis* Diverse Impact on the Human Immune System. Int. J. Mol. Sci..

[11] Khan V., Putluri N., Sreekumar A., Mindikoglu A.L. (2018). Current Applications of Metabolomics in Cirrhosis. Metabolites.

[12] Franco-Duarte R., Černáková L., Kadam S., Kaushik K.S., Salehi B., Bevilacqua A., Corbo M.R., Antolak H., Dybka-Stępień K., Leszczewicz M. (2019). Advances in Chemical and Biological Methods to Identify Microorganisms-From Past to Present. Microorganisms.

[13] Kubina R., Dziedzic A. (2020). Molecular and Serological Tests for COVID-19 a Comparative Review of SARS-CoV-2 Coronavirus Laboratory and Point-of-Care Diagnostics. Diagnostics.

[14] Lee R.A., Al Dhaheri F., Pollock N.R., Sharma T.S. (2020). Assessment of the Clinical Utility of Plasma Metagenomic Next-Generation Sequencing in a Pediatric Hospital Population. J. Clin. Microbiol..

[15] Rebrosova K., Samek O., Kizovsky M., Bernatova S., Hola V., Ruzicka F. (2022). Raman Spectroscopy-A Novel Method for Identification and Characterization of Microbes on a Single-Cell Level in Clinical Settings. Front. Cell Infect. Microbiol..

[16] Perala R.S., Chandrasekar N., Balaji R., Alexander P.S., Humaidi N.Z.N., Hwang M.T. (2024). A comprehensive review on graphene-based materials: From synthesis to contemporary sensor applications. Mater. Sci. Eng. R Rep..

[17] Naman A., Tahseen H., Nawaz H., Majeed M.I., Ali A., Haque A., Akbar M.U., Mehmood N., Nosheen R., Nadeem S. (2024). Surface-enhanced Raman spectroscopy for characterization of supernatant samples of biofilm forming bacterial strains. Spectrochim. Acta Part A Mol. Biomol. Spectrosc..

[18] Dmitrieva E.V., Kapitanova O.O., Lv S., Sinyashin O.G., Veselova I.A. (2025). Coupling of chromatography with surface-enhanced Raman spectroscopy: Trends and prospects. Front. Chem..

[19] Usoltsev D., Sitnikova V., Kajava A., Uspenskaya M. (2019). Systematic FTIR Spectroscopy Study of the Secondary Structure Changes in Human Serum Albumin under Various Denaturation Conditions. Biomolecules.

[20] Pezzotti G. (2021). Raman spectroscopy in cell biology and microbiology. J. Raman Spectrosc..

[21] Azan A., Untereiner V., Gobinet C., Sockalingum G.D., Breton M., Piot O., Mir L.M. (2017). Demonstration of the Protein Involvement in Cell Electropermeabilization using Confocal Raman Microspectroscopy. Sci. Rep..

[22] Lee D., Hussain S., Yeo J., Pang Y. (2021). Adsorption of dipeptide L-alanyl-L-tryptophan on gold colloidal nanoparticles studied by surface-enhanced Raman spectroscopy. Spectrochim. Acta Part A Mol. Biomol. Spectrosc..

[23] Chen P., Zhang F., Lin L., Bai H., Zhang L., Tang G.Q., Fang H., Mu G.G., Gong W., Liu Z.P. (2011). Raman Spectroscopy for Noninvasive Monitoring of Umbilical Cord Mesenchymal Stem Cells Viability Transitions. Stem Cells in Clinic and Research.

[24] Okotrub K.A., Surovtsev N.V., Semeshin V.F., Omelyanchuk L.V. (2015). Raman spectroscopy for DNA quantification in cell nucleus. Cytometry.

[25] Matthäus C., Bird B., Miljković M., Chernenko T., Romeo M., Diem M. (2008). Chapter 10: Infrared and Raman microscopy in cell biology. Methods Cell Biol..

[26] Baron V.O., Chen M., Hammarstrom B., Hammond R.J.H., Glynne-Jones P., Gillespie S.H., Dholakia K. (2020). Real-time monitoring of live mycobacteria with a microfluidic acoustic-Raman platform. Commun. Biol..

[27] Klingler S., Hniopek J., Stach R., Schmitt M., Popp J., Mizaikoff B. (2021). Simultaneous Infrared Spectroscopy, Raman Spectroscopy, and Luminescence Sensing: A Multispectroscopic Analytical Platform. ACS Meas. Sci. Au.

[28] Miura T., Takeuchi H., Harada I. (1988). Characterization of individual tryptophan side chains in proteins using Raman spectroscopy and hydrogen-deuterium exchange kinetics. Biochemistry.

[29] Safar W., Azziz A., Edely M., Lamy de la Chapelle M. (2023). Conventional Raman, SERS and TERS Studies of DNA Compounds. Chemosensors.

[30] Siamwiza M.N., Lord R.C., Chen M.C., Takamatsu T., Harada I., Matsuura H., Shimanouchi T. (1975). Interpretation of the doublet at 850 and 830 cm-1 in the Raman spectra of tyrosyl residues in proteins and certain model compounds. Biochemistry.

[31] Hilton K.L.F., Manwani C., Boles J.E., White L.J., Ozturk S., Garrett M.D., Hiscock J.R. (2021). The phospholipid membrane compositions of bacterial cells, cancer cell lines and biological samples from cancer patients. Chem. Sci..

[32] Golichenko B. (2017). Raman study of L-Asparagine and L-Glutamine molecules adsorbed on aluminum films in a wide frequency range. Semicond. Phys. Quantum Electron. Optoelectron..

[33] Cheuquepan W., Hernandez S., Perez-Estebanez M., Romay L., Heras A., Colina A. (2021). Electrochemical generation of surface enhanced Raman scattering substrates for the determination of folic acid. J. Electroanal. Chem..

[34] Jung T.S., Hahm J.R., Kim J.J., Jung J.H., Kang M.Y., Moon S.W., Lee K.W., Kim H.C., Lee J.D., Kim J.H. (2005). Determination of urinary Myo-/chiro-inositol ratios from Korean diabetes patients. Yonsei Med. J..

[35] Aboueimehrizi E., Makaremy M.A., Bazrpash S., Noormohammadi F., Darestani Y.R., Nourany M. (2022). Synthesis of high-modulus thermoset PUs of PCL-PTMG/CNW Biomaterials with different Soft Domain Architecture and Composition for high Shape Memory Performance. Cellulose.

[36] https://www.biolot.ru/catalogue/Cell_culture_media/Minimum_Essential_Medium_Eagle_(MEM)_Alpha_Modification_with_L-glutamine_liquid_sterile-filtered_PET_450_ml.html.

[37] Burikov S., Dolenko T.A., Fadeev V., Sugonyaev A. (2007). Identification of inorganic salts and determination of their concentrations in aqueous solutions based on the valence Raman band of water using artificial neural networks. Pattern Recognit. Image Anal..

[38] Loyola-Leyva A., Hernández-Vidales K., Loyola-Rodríguez J.P., González F.J. (2024). Noninvasive Glucose Measurements Through Transcutaneous Raman Spectroscopy: A Review. J. Diabetes Sci. Technol..

[39] Sun C., Guo N., Ye L., Miao L., Cao M., Yan M., Ding J. (2023). Quantitative detection of phenol red by surface enhanced Raman spectroscopy based on improved GA-BP. Spectrochim. Acta Part A Mol. Biomol. Spectrosc..

[40] Zhang X., Niu P., Liu L., Ye T., Ding W., Wei X., Zhu T., Li Z., Fang H., Liu H. (2025). Multivariate Modular Metabolic Engineering for the Enhanced Biosynthesis of Cytidine in *Bacillus subtilis*. J. Agric. Food Chem..

[41] Hernández B., Coïc Y.M., Kruglik S.G., Sanchez-Cortes S., Ghomi M. (2024). The relationship between the tyrosine residue 850–830 cm^−1^ Raman doublet intensity ratio and the aromatic side chain χ_1_ torsion angle. Spectrochim. Acta Part A Mol. Biomol. Spectrosc..

[42] Näpflin N., Schubert C., Malfertheiner L., Hardt W.D., von Mering C. (2025). Gene-level analysis of core carbohydrate metabolism across the *Enterobacter*iaceae pan-genome. Commun. Biol..

